# Breaking Barriers: A Single-Center Real-World Experience With Recruitment and Administration of COVID-19 Monoclonal Antibodies and Opportunities for Improvement

**DOI:** 10.1093/ofid/ofad665

**Published:** 2023-12-26

**Authors:** Sara Brenner, Alexander Knee, Douglas Salvador, Erica Housman, Gladys Fernandez, Armando Paez

**Affiliations:** Division of Infectious Diseases, University of California, San Diego, San Diego, California, USA; Department of Medicine, and Epidemiology/Biostatistics Research Core, UMass Chan Medical School–Baystate, Springfield, Massachusetts, USA; Division of Infectious Diseases, UMass Chan Medical School–Baystate, Springfield, Massachusetts, USA; Department of Pharmacy, UMass Chan Medical School–Baystate, Springfield, Massachusetts, USA; Department of Surgery, UMass Chan Medical School–Baystate, Springfield, Massachusetts, USA; Division of Infectious Diseases, UMass Chan Medical School–Baystate, Springfield, Massachusetts, USA

**Keywords:** COVID-19, health disparities, monoclonal antibodies, Social Vulnerability Index (SVI), pandemic

## Abstract

We conducted a retrospective exploratory study evaluating factors associated with selection to receive and infusion with coronavirus disease 2019 monoclonal antibodies. While priority was given to high-risk patients, patients with increased Social Vulnerability Index scores were less likely to present for infusion, raising concern that social factors created barriers to treatment.

Coronavirus disease 2019 (COVID-19) monoclonal antibodies (mAbs) have been shown to be effective in reducing the risk of emergency room visits and hospitalizations in patients who are at high risk for disease progression [1, 2]. Significant disparities have been noted in the outcomes of patients infected with COVID-19 [3, 4]. Multiple studies have shown that patients who identify as Black or Hispanic/Latinx or whose primary language is not English were less likely to receive mAbs [5–8]. However, there has been limited study of the barriers that prevented these patients from accessing treatment. One survey noted that non-White participants reported less awareness of mAbs than White participants, but there was no significant difference in whether they thought they would accept treatment if they were to become infected [9]. There are limited data about the characteristics of patients who chose not to pursue treatment [10] and none to our knowledge about the perceptions of those who were referred but chose not to pursue treatment.

## METHODS

This was an exploratory retrospective cohort study of patients who were referred for mAbs treatment from 7 December 2020 to 20 May 2021. The aim of the study was to describe the characteristics of patients who were selected for and those ultimately infused with mAbs under the Emergency Use Authorization (EUA) early in the COVID-19 pandemic in order to generate hypotheses of potential barriers to care. At the time of the study, mAbs were administered with guidance from the Massachusetts Department of Public Health (DPH), which devised an allocation strategy to attempt to reach patients with the highest risk of severe infection and distribute mAbs equitably. DPH allocated mAbs to centers based on the case rate, hospitalization rate, and availability.

Per DPH recommendations, healthcare systems were to allocate available doses in order to “prioritize patients aged ≥65 years and those aged ≥18 years with BMI [body mass index] ≥35 (Tier 1) over others who meet EUA criteria (Tier 2)” and if the number of patients exceeded the supply of available doses, as in the case of our system, “hospitals should determine access through a lottery system with 20% of units set aside for vulnerable populations.” Social vulnerability was assessed using the Social Vulnerability Index (SVI) for the patient's home address. SVI is a measure devised by the Centers for Disease Control and Prevention that assigns a score to census tracts in order to identify communities that are at the highest risk for adverse outcomes in the event of a disaster such as a pandemic. Per DPH guidelines, our system employed a lottery system in which 20% of mAbs were reserved for patients living in a census tract with SVI >0.5 or a city/town with 7-day average COVID-19 incidence rate in the top quartile. For the remaining 80%, preference was given to patients meeting Tier 1 criteria followed by patients meeting Tier 2 criteria. Patients who were selected via the lottery system were contacted by the infusion center team and scheduled for infusion, at which time some patients no longer qualified for infusion and some declined therapy. Throughout the study, patients were infused with the mAbs available at that time.

For the purposes of our study, we reviewed all patients who were referred for mAbs therapy. Inclusion criteria for the study required symptom onset within 10 days of referral, high risk for severe disease based on criteria outlined by EUA/DPH, and follow-up performed by an in-network primary care provider in order to be able to evaluate preexisting diagnoses and demographics and to track their treatment course. Patients were excluded if they were hospitalized or meeting criteria for hospitalization. Predictors of interest were obtained from the initial referral documentation and through medical record review. For patients who were selected for treatment but did not undergo infusion, reasons were documented and categorized as either disqualified or declining treatment.

### Statistical Analysis

Continuous variables were summarized using mean and standard deviation (SD), and/or median and percentile. Categorical variables were summarized using frequencies and percentages. Associations between predictors and our outcomes were estimated using unadjusted logistic regression and reported as absolute percentage point differences and 95% confidence intervals (CIs). We considered predictors with at least a ±10% absolute difference to suggest possibly clinically relevant associations for future study. Analysis was conducted using Stata MP, version 17.0 (StataCorp LLC, College Station, Texas).

### Patient Consent

This study was reviewed, approved, and exempted by the Baystate Medical Center Institutional Review Board. No patient consent was required to conduct the study.

## RESULTS

### Study Population

Of 285 patients referred for mAbs infusion, 230 patients met inclusion criteria. The primary reason for exclusion was referral from outside of network. In the study population, 40% were male, and the mean age was 60 (SD, 15) years. Most patients reported an English language preference (87%), with the second most common language preference being Spanish (10%). A total of 151 (66%) identified as White, non-Hispanic and 46% had SVI >0.50. Most patients had 1–2 comorbid conditions (53%), with an additional 37% having 3 or more comorbid conditions. There were 179 patients (78%) selected for infusion via the lottery system, of which 119 (66%) were infused.

### Selection and Infusion

**Figure 1.**
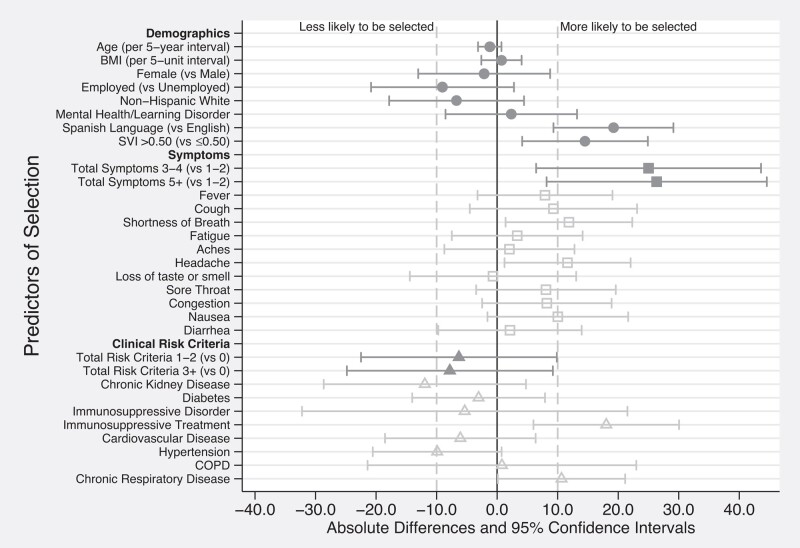
Baseline characteristics associated with selection for monoclonal antibody infusion. Patient characteristics with at least ± 10 percentage point differences (represented by the vertical dashed lines) were considered to have a possible association with selection for monoclonal antibody infusion. Abbreviations: BMI, body mass index; COPD, chronic obstructive pulmonary disease; SVI, Social Vulnerability Index.

Among eligible patients who were referred (n = 179), patients selected were more likely to report Spanish language preference (19% more likely to be selected), have SVI >0.50 (14.5% more likely), and have higher symptom count (3–4 symptoms, 25% more likely; ≥5 total symptoms, 26% more likely). Specific symptoms associated with selection included shortness of breath (12% more likely), headache (12% more likely), and nausea/vomiting (10% more likely). Those with chronic kidney disease or hypertension were 12% and 10% less likely to be selected, respectively. However, those receiving immunosuppressive treatment or with chronic respiratory disease were 18% and 11% more likely to be selected (Figure 1, Supplementary Material 1 and 2).

**Figure 2.**
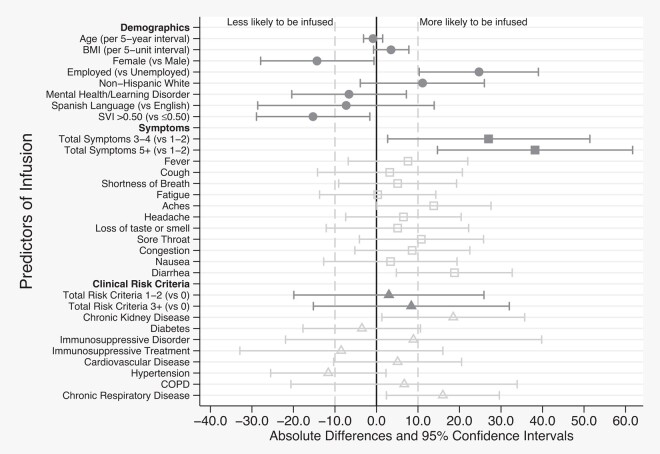
Baseline characteristics associated with infusion with monoclonal antibody. Patient characteristics with at least ± 10 percentage point differences (represented by the vertical dashed lines) were considered to have a possible association with infusion with monoclonal antibody. Abbreviations: BMI, body mass index; COPD, chronic obstructive pulmonary disease; SVI, Social Vulnerability Index.

Among those who ultimately presented for infusion (n = 119), we note that higher percentages were male (14% higher), were employed (25% higher), and identified as non-Hispanic White (11% higher). In addition, while a higher percentage of those living in communities with an SVI >0.50 were selected, they were 15% less likely to be infused. Like selection, those with a higher total number of symptoms were more likely to be infused (3–4 symptoms, 27% more likely; ≥5 total symptoms, 38% more likely vs 1–2 symptoms). Symptoms associated with infusion included body aches (14% more likely), sore throat (11% more likely), and diarrhea (19% more likely). Total number of comorbid conditions was not associated with selection or infusion. However, specific risk criteria were. Those with chronic kidney and respiratory diseases were 19% and 16% more likely to be infused, respectively (Figure 2, Supplementary Material 3 and 4).

### Reasons Not Infused

Of the 60 patients who were selected but not infused, 19 (32%) became ineligible, 39 (65%) declined treatment, and 2 (3%) were not infused for unclear reasons. Reasons for ineligibility were either clinical decompensation (n = 10 [17%]) or passing the 10-day treatment window at the time of selection (n = 9 [15%]). However, patients were generally able to present within the recommended 10-day timeframe following symptom onset, with average time from symptom onset to infusion ranging from 5.4 to 7.1 days (Supplementary Material 5) over the study period and only 9 (5% of those selected) passing the treatment window. Of the patients who declined treatment, 12 (20%) reported they no longer desired treatment due to clinical improvement, 6 (10%) reported concerns about mAbs safety, 4 (7%) were unable to find a caregiver for a family member, 3 (5%) reported lack of transportation, and 2 (3%) reported that the timing of the infusion was inconvenient. Some reported >1 barrier. Unfortunately, we could not discern reasons for declining treatment in 22% of these patients.

## DISCUSSION

Our study highlights some successes and challenges of administering mAbs early in the pandemic. Rapid implementation of mAbs therapy demonstrates an approach to mobilizing resources quickly during a disaster at a time when there was limited information, no definitive treatment, and few diagnostic and therapeutic resources relative to the number of cases. It is unclear how changes in recommended timing for mAbs treatment would have affected this, especially with changes in testing, public perception of COVID-19, and availability of alternative treatments and vaccines.

The main concerns raised in our study were disparities among patients who were infused. While the patients who were believed to be most vulnerable based on SVI were more likely to be selected, they were ultimately less likely to receive mAbs. Past studies identified disparities in mAbs treatment among racial/ethnic groups [5–8, 10]. Our findings similarly demonstrate that when compared to patients identifying as White and non-Hispanic, patients who identified as non-White or Hispanic were more likely to be selected for mAbs infusion but less likely to complete infusion. Disparities were also found in gender, language, and employment status. Among the cited reasons for declining treatment, most commonly, patients cited feeling better and thought it was no longer necessary. It is challenging to assess disparities in the availability of education and informational resources within the study population and how they might have impacted the likelihood of mAbs infusion. Additionally, some patients also reported a lack of transportation or alternative caregivers for family members, which may relate to socioeconomic status and the locations in which they reside. Transportation was offered throughout the study, but it is unknown how patients perceived this resource. The proximity of the mAbs clinic may also have impacted patients’ access as the clinic location was moved approximately 13 miles from the original site in March 2021 in order to accommodate a larger patient volume. The new site was within the same county, but the original location was in a city with lower median household income and higher unemployment rate [11]. While the barriers reported in the study may explain some of the disparities observed, 22% of those who declined treatment did not have a documented reason. There may also be additional barriers not readily captured in the data.

There have been some attempts to improve access to mAbs through mobile units [12, 13] and same-day infusion at a community clinic [14], but it is unclear whether these interventions could be generalized to other systems, especially those with more limited resources. While there were no oral therapies available at the time of this study, racial/ethnic disparities exist in the administration of oral therapies as well [10], suggesting that the logistics of accessing mAbs infusion are not the only barriers to care. Our findings that patients with higher SVI, women, racial/ethnic minorities, patients who were unemployed, and patients whose primary language was not English were less likely to receive mAbs treatment emphasize the need for improving access to care. Further study is needed to better characterize the barriers to treatment and facilitate improved access for these groups, whether for future treatment with mAbs or other novel therapeutics.

## Supplementary Material

ofad665_Supplementary_Data

## Supplementary Data

Supplementary materials are available at *Open Forum Infectious Diseases* online. Consisting of data provided by the authors to benefit the reader, the posted materials are not copyedited and are the sole responsibility of the authors, so questions or comments should be addressed to the corresponding author.

## Notes

***Potential conflicts of interest.*** A. P. receives research support from AstraZeneca paid to Baystate Medical Center. All other authors report no potential conflicts.
