# Supplementary material for: Breaking Barriers: A Single-Center Real-World Experience With Recruitment and Administration of COVID-19 Monoclonal Antibodies and Opportunities for Improvement
Source: Open Forum Infect Dis. 2023 Dec 26;11(2):ofad665. doi: 10.1093/ofid/ofad665 (PMC10847809; doi:10.1093/ofid/ofad665)

# **Supplement 1. Baseline characteristics associated with selection for monoclonal antibody infusion**

|  | **Overall** | **Never Selected** | **Selected** | **Absolute Difference** |
| --- | --- | --- | --- | --- |
|  | **(N = 230)** | **(N = 51)** | **(N = 179)** | **(95% CI)** |
|  |  |  |  |  |
| Age at Referral: Mean (SD) | 60.2 (14.6) | 62.5 (11.0) | 59.6 (15.4) | -1.2% (-3.2 to 0.7)^b^ |
| BMI: Mean (SD) | 34.6 (8.2) | 34.2 (7.7) | 34.7 (8.4) | 0.7% (-2.6 to 4.0)^b^ |
| Sex: n(%) |  |  |  |  |
| Male | 91 (39.6%) | 19 (20.9%) | 72 (79.1%) | Referent |
| Female | 139 (60.4%) | 32 (23.0%) | 107 (77.0%) | -2.1% (-13.0 to 8.8) |
| Employment: n(%) |  |  |  |  |
| Unemployed | 89 (38.7%) | 16 (18.0%) | 73 (82.0%) | Referent |
| Employed | 100 (43.5%) | 27 (27.0%) | 73 (73.0%) | -9.0% (-20.8 to 2.8) |
| *Missing* | *41 (17.8%)* | 8 (19.5%) | 33 (80.5%) | *-* |
| Race/Ethnicity: n(%) |  |  |  |  |
| Non-White or Hispanic | 73 (31.7%) | 13 (17.8%) | 60 (82.2%) | Referent |
| White Non-Hispanic | 151 (65.7%) | 37 (24.5%) | 114 (75.5%) | -6.7 (-17.8 to 4.4) |
| *Missing* | *6 (2.6%)* | 1 (16.7%) | 5 (83.3%) | *-* |
| Mental Health/Learning Disorder: n(%) |  |  |  |  |
| No | 103 (44.8%) | 24 (23.3%) | 79 (76.7%) | Referent |
| Yes | 124 (53.9%) | 26 (21.0%) | 98 (79.0%) | 2.3% (-8.5 to 13.2) |
| Choose not to answer | 3 (1.3%) | 1 (33.3%) | 2 (66.7%) | - |
| Preferred Language: n(%) |  |  |  |  |
| English | 201 (87.4%) | 47 (23.4%) | 154 (76.6%) | Referent |
| Spanish | 24 (10.4%) | 1 (4.2%) | 23 (95.8%) | 19.2% (9.3 to 29.1) |
| Other | 4 (1.7%) | 3 (75.0%) | 1 (25.0%) | - |
| *Missing* | *1 (0.4%)* | 0 (0.0%) | 1 (100.0%) | *-* |
| Social Vulnerability Index: n(%) |  |  |  |  |
| ≤0.50 | 125 (54.3%) | 36 (28.8%) | 89 (71.2%) | Referent |
| >0.50 | 105 (45.7%) | 15 (14.3%) | 90 (85.7%) | 14.5% (4.1 to 24.9) |
| Total Symptoms at Referral: n(%) |  |  |  |  |
| 1-2 | 34 (14.8%) | 15 (44.1%) | 19 (55.9%) | Referent |
| 3-4 | 89 (38.7%) | 17 (19.1%) | 72 (80.9%) | 25.0% (6.4 to 43.6) |
| 5+ | 107 (46.5%) | 19 (17.8%) | 88 (82.2%) | 26.4% (8.2 to 44.6) |
| Total Risk Criteria at Referral: n(%) |  |  |  |  |
| 0 factors | 25 (10.9%) | 4 (16.0%) | 21 (84.0%) | Referent |
| 1-2 factors | 121 (52.6%) | 27 (22.3%) | 94 (77.7%) | -6.3% (-22.5 to 9.9) |
| 3+ factors | 84 (36.5%) | 20 (23.8%) | 64 (76.2%) | -7.8% (-24.8 to 9.2) |
|  |  |  |  |  |

# **Supplement 2. Symptoms and risk criteria associated with selection for monoclonal antibody infusion**

|  | **Overall** | **Never Selected** | **Selected** |  |
| --- | --- | --- | --- | --- |
|  | **(N = 230)** | **(N = 51)** | **(N = 179)** | **Absolute Difference (95% CI)** |
| **Symptoms** |  |  |  |  |
| Total Number of Symptoms At Referral |  |  |  |  |
| Mean (SD) | 4.4 (1.9) | 3.7 (1.7) | 4.6 (1.9) | - |
| Median (Q1, Q3) | 4.0 (3.0, 6.0) | 4.0 (2.0, 5.0) | 4.0 (3.0, 6.0) | - |
| Min, Max | 1.0, 10.0 | 1.0, 8.0 | 1.0, 10.0 | - |
| Fever or Chills |  |  |  |  |
| No | 93 (40.4%) | 25 (26.9%) | 68 (73.1%) | Referent |
| Yes | 137 (59.6%) | 26 (19.0%) | 111 (81.0%) | 7.9% (-3.2 to 19.1) |
| Cough |  |  |  |  |
| No | 51 (22.2%) | 15 (29.4%) | 36 (70.6%) | Referent |
| Yes | 179 (77.8%) | 36 (20.1%) | 143 (79.9%) | 9.3% (-4.5 to 23.1) |
| Shortness of Breath or Difficulty Breathing |  |  |  |  |
| No | 153 (66.5%) | 40 (26.1%) | 113 (73.9%) | Referent |
| Yes | 77 (33.5%) | 11 (14.3%) | 66 (85.7%) | 11.9% (1.4 to 22.3) |
| Fatigue |  |  |  |  |
| No | 136 (59.1%) | 32 (23.5%) | 104 (76.5%) | Referent |
| Yes | 94 (40.9%) | 19 (20.2%) | 75 (79.8%) | 3.3% (-7.5 to 14.1) |
| Muscle or Body Aches |  |  |  |  |
| No | 112 (48.7%) | 26 (23.2%) | 86 (76.8%) | Referent |
| Yes | 118 (51.3%) | 25 (21.2%) | 93 (78.8%) | 2.0% (-8.7 to 12.8) |
| Headache |  |  |  |  |
| No | 143 (62.2%) | 38 (26.6%) | 105 (73.4%) | Referent |
| Yes | 87 (37.8%) | 13 (14.9%) | 74 (85.1%) | 11.6% (1.2 to 22.0) |
| New Loss of Taste or Smell |  |  |  |  |
| No | 186 (80.9%) | 41 (22.0%) | 145 (78.0%) | Referent |
| Yes | 44 (19.1%) | 10 (22.7%) | 34 (77.3%) | -0.7% (-14.4 to 13.1) |
| Sore Throat |  |  |  |  |
| No | 174 (75.7%) | 42 (24.1%) | 132 (75.9%) | Referent |
| Yes | 56 (24.3%) | 9 (16.1%) | 47 (83.9%) | 8.1% (-3.5 to 19.6) |
| Congestion or Runny Nose |  |  |  |  |
| No | 114 (49.6%) | 30 (26.3%) | 84 (73.7%) | Referent |
| Yes | 116 (50.4%) | 21 (18.1%) | 95 (81.9%) | 8.2% (-2.5 to 18.9) |
| Nausea or Vomiting |  |  |  |  |
| No | 181 (78.7%) | 44 (24.3%) | 137 (75.7%) | Referent |
| Yes | 49 (21.3%) | 7 (14.3%) | 42 (85.7%) | 10.0% (-1.6 to 21.6) |
| Diarrhea |  |  |  |  |
| No | 167 (72.6%) | 38 (22.8%) | 129 (77.2%) | Referent |
| Yes | 63 (27.4%) | 13 (20.6%) | 50 (79.4%) | 2.1% (-9.7 to 14.0) |
| **Risk Criteria** |  |  |  |  |
| Total Number of Risk Criteria |  |  |  |  |
| Mean (SD) | 2.1 (1.4) | 2.2 (1.4) | 2.1 (1.4) | - |
| Median (Q1, Q3) | 2.0 (1.0, 3.0) | 2.0 (1.0, 3.0) | 2.0 (1.0, 3.0) | - |
| Min, Max | 0.0, 5.0 | 0.0, 5.0 | 0.0, 5.0 | - |
| Chronic Kidney Disease |  |  |  |  |
| No | 196 (85.2%) | 40 (20.4%) | 156 (79.6%) | Referent |
| Yes | 34 (14.8%) | 11 (32.4%) | 23 (67.6%) | -11.9% (-24.8 to 9.2) |
| Diabetes |  |  |  |  |
| No | 134 (58.3%) | 28 (20.9%) | 106 (79.1%) | Referent |
| Yes | 96 (41.7%) | 23 (24.0%) | 73 (76.0%) | -3.1% (-14.0 to 7.9) |
| Immunosuppressive Disease |  |  |  |  |
| No | 219 (95.2%) | 48 (21.9%) | 171 (78.1%) | Referent |
| Yes | 11 (4.8%) | 3 (27.3%) | 8 (72.7%) | -5.4% (-32.2 to 21.5) |
| Receiving Immunosuppressive Treatment |  |  |  |  |
| No | 212 (92.2%) | 50 (23.6%) | 162 (76.4%) | Referent |
| Yes | 18 (7.8%) | 1 (5.6%) | 17 (94.4%) | 18.0% (6.0 to 30.1) |
| Cardiovascular Disease |  |  |  |  |
| No | 166 (72.2%) | 34 (20.5%) | 132 (79.5%) | Referent |
| Yes | 64 (27.8%) | 17 (26.6%) | 47 (73.4%) | -6.1% (-18.5 to 6.4) |
| Hypertension |  |  |  |  |
| No | 77 (33.5%) | 12 (15.6%) | 65 (84.4%) | Referent |
| Yes | 153 (66.5%) | 39 (25.5%) | 114 (74.5%) | -9.9% (-20.6 to 0.7) |
| COPD |  |  |  |  |
| No | 216 (93.9%) | 48 (22.2%) | 168 (77.8%) | Referent |
| Yes | 14 (6.1%) | 3 (21.4%) | 11 (78.6%) | 0.8% (-21.4 to 23.0) |
| Chronic Respiratory Disease |  |  |  |  |
| No | 126 (54.8%) | 34 (27.0%) | 92 (73.0%) | Referent |
| Yes | 104 (45.2%) | 17 (16.3%) | 87 (83.7%) | 10.6% (0.1 to 21.2) |
|  |  |  |  |  |

**Supplement 3. Baseline characteristics associated with infusion with monoclonal antibody**

|  | **Overall** | **Not Infused** | **Infused** | **Absolute Difference** |
| --- | --- | --- | --- | --- |
|  | **(N = 179)** | **(N = 60)** | **(N = 119)** | **(95% CI)** |
| Age at Referral: Mean (SD) |  |  |  |  |
|  | 59.6 (15.4) | 60.7 (16.4) | 59.1 (14.9) | -0.8% (-3.1 to 1.5)^b^ |
| BMI: Mean (SD) |  |  |  |  |
|  | 34.7 (8.4) | 33.2 (7.9) | 35.4 (8.6) | 3.5% (-0.7 to 7.8)^b^ |
| Sex: n(%) |  |  |  |  |
| Male | 72 (40.2%) | 18 (25.0%) | 54 (75.0%) | Referent |
| Female | 107 (59.8%) | 42 (39.3%) | 65 (60.7%) | -14.3% (-27.9 to -0.6) |
| Employment: n(%) |  |  |  |  |
| Unemployed | 73 (40.8%) | 31 (42.5%) | 42 (57.5%) | Referent |
| Employed | 73 (40.8%) | 13 (17.8%) | 60 (82.2%) | 24.7% (10.3 to 39.0) |
| *Missing* | *33 (18.4%)* | *16 (48.5%)* | *17 (51.5%)* | - |
| Race/Ethnicity: n(%) |  |  |  |  |
| Non-White or Hispanic | 60 (33.5%) | 24 (40.0%) | 36 (60.0%) | Referent |
| White Non-Hispanic | 114 (63.7%) | 33 (28.9%) | 81 (71.1%) | 11.1% (-3.9 to 26.0) |
| *Missing* | *5 (2.8%)* | *3 (60.0%)* | *2 (40.0%)* | *-* |
| Mental Health/Learning Disorder: n(%) |  |  |  |  |
| No | 79 (44.1%) | 23 (29.1%) | 56 (70.9%) | Referent |
| Yes | 98 (54.7%) | 35 (35.7%) | 63 (64.3%) | -6.6% (-20.4 to 7.2) |
| Choose not to answer | 2 (1.1%) | 2 (100.0%) | 0 (0.0%) | - |
| Preferred Language: n(%) |  |  |  |  |
| English | 154 (86.0%) | 49 (31.8%) | 105 (68.2%) | Referent |
| Spanish | 23 (12.8%) | 9 (39.1%) | 14 (60.9%) | -7.3% (-28.6 to 13.9) |
| Other | 1 (0.6%) | 1 (100.0%) | 0 (0.0%) | - |
| *Missing* | *1 (0.6%)* | *1 (100.0%)* | *0 (0.0%)* | *-* |
| Social Vulnerability Index: n(%) |  |  |  |  |
| ≤0.50 | 89 (49.7%) | 23 (25.8%) | 66 (74.2%) | Referent |
| >0.50 | 90 (50.3%) | 37 (41.1%) | 53 (58.9%) | -15.3% (-28.9 to -1.6) |
| Total Symptoms at Referral: n(%) |  |  |  |  |
| 1-2 | 19 (10.6%) | 12 (63.2%) | 7 (36.8%) | Referent |
| 3-4 | 72 (40.2%) | 26 (36.1%) | 46 (63.9%) | 27.0% (2.7 to 51.4) |
| 5+ | 88 (49.2%) | 22 (25.0%) | 66 (75.0%) | 38.2% (14.7 to 61.7) |
| Total Risk Criteria at Referral: n(%) |  |  |  |  |
| 0 factors | 21 (11.7%) | 8 (38.1%) | 13 (61.9%) | Referent |
| 1-2 factors | 94 (52.5%) | 33 (35.1%) | 61 (64.9%) | 3.0% (-19.9 to 25.9) |
| 3+ factors | 64 (35.8%) | 19 (29.7%) | 45 (70.3%) | 8.4% (-15.2 to 32.0) |
|  |  |  |  |  |
|  |  |  |  |  |

# **Supplement 4. Symptoms and risk criteria associated with infusion with monoclonal antibody**

|  | **Overall** | **Not Infused** | **Infused** |  |
| --- | --- | --- | --- | --- |
|  | **(N = 179)** | **(N = 60)** | **(N = 119)** | **Absolute Difference (95% CI)** |
| **Symptoms** |  |  |  |  |
| Total Number of Symptoms at Referral |  |  |  |  |
| Mean (SD) | 4.6 (1.9) | 4.1 (1.9) | 4.9 (1.8) | - |
| Median (Q1, Q3) | 4.0 (3.0, 6.0) | 4.0 (3.0, 5.0) | 5.0 (4.0, 6.0) | - |
| Min, Max | 1.0, 10.0 | 1.0, 10.0 | 1.0, 9.0 | - |
| Fever or Chills |  |  |  |  |
| No | 68 (38.0%) | 26 (38.2%) | 42 (61.8%) | Referent |
| Yes | 111 (62.0%) | 34 (30.6%) | 77 (69.4%) | 7.6% (-6.8 to 22.0) |
| Cough |  |  |  |  |
| No | 36 (20.1%) | 13 (36.1%) | 23 (63.9%) | Referent |
| Yes | 143 (79.9%) | 47 (32.9%) | 96 (67.1%) | 3.2% (-14.2 to 20.7) |
| Shortness of Breath or Difficulty Breathing |  |  |  |  |
| No | 113 (63.1%) | 40 (35.4%) | 73 (64.6%) | Referent |
| Yes | 66 (36.9%) | 20 (30.3%) | 46 (69.7%) | 5.1% (-9.1 to 19.3) |
| Fatigue |  |  |  |  |
| No | 104 (58.1%) | 35 (33.7%) | 69 (66.3%) | Referent |
| Yes | 75 (41.9%) | 25 (33.3%) | 50 (66.7%) | 0.3% (-13.7 to 14.3) |
| Muscle or Body Aches |  |  |  |  |
| No | 86 (48.0%) | 35 (40.7%) | 51 (59.3%) | Referent |
| Yes | 93 (52.0%) | 25 (26.9%) | 68 (73.1%) | 13.8% (0.1 to 27.6) |
| Headache |  |  |  |  |
| No | 105 (58.7%) | 38 (36.2%) | 67 (63.8%) | Referent |
| Yes | 74 (41.3%) | 22 (29.7%) | 52 (70.3%) | 6.5% (-7.4 to 20.4) |
| New Loss of Taste or Smell |  |  |  |  |
| No | 145 (81.0%) | 50 (34.5%) | 95 (65.5%) | Referent |
| Yes | 34 (19.0%) | 10 (29.4%) | 24 (70.6%) | 5.1% (-12.1 to 22.2) |
| Sore Throat |  |  |  |  |
| No | 132 (73.7%) | 48 (36.4%) | 84 (63.6%) | Referent |
| Yes | 47 (26.3%) | 12 (25.5%) | 35 (74.5%) | 10.8% (-4.1 to 25.8) |
| Congestion or Runny Nose |  |  |  |  |
| No | 84 (46.9%) | 32 (38.1%) | 52 (61.9%) | Referent |
| Yes | 95 (53.1%) | 28 (29.5%) | 67 (70.5%) | 8.6% (-5.2 to 22.5) |
| Nausea or Vomiting |  |  |  |  |
| No | 137 (76.5%) | 47 (34.3%) | 90 (65.7%) | Referent |
| Yes | 42 (23.5%) | 13 (31.0%) | 29 (69.0%) | 3.4% (-12.7 to 19.4) |
| Diarrhea |  |  |  |  |
| No | 129 (72.1%) | 50 (38.8%) | 79 (61.2%) | Referent |
| Yes | 50 (27.9%) | 10 (20.0%) | 40 (80.0%) | 18.8% (4.8 to 32.7) |
| **Risk Criteria** |  |  |  |  |
| Total Number of Risk Criteria |  |  |  |  |
| Mean (SD) | 2.1 (1.4) | 2.0 (1.3) | 2.2 (1.4) | - |
| Median (Q1, Q3) | 2.0 (1.0, 3.0) | 2.0 (1.0, 3.0) | 2.0 (1.0, 3.0) | - |
| Min, Max | 0.0, 5.0 | 0.0, 5.0 | 0.0, 5.0 | - |
| Chronic Kidney Disease |  |  |  |  |
| No | 156 (87.2%) | 56 (35.9%) | 100 (64.1%) | Referent |
| Yes | 23 (12.8%) | 4 (17.4%) | 19 (82.6%) | 18.5% (1.3 to 35.7) |
| Diabetes |  |  |  |  |
| No | 106 (59.2%) | 34 (32.1%) | 72 (67.9%) | Referent |
| Yes | 73 (40.8%) | 26 (35.6%) | 47 (64.4%) | -3.5% (-17.7 to 10.6) |
| Immunosuppressive Disease |  |  |  |  |
| No | 171 (95.5%) | 58 (33.9%) | 113 (66.1%) | Referent |
| Yes | 8 (4.5%) | 2 (25.0%) | 6 (75.0%) | 8.9% (-21.9 to 39.8) |
| Receiving Immunosuppressive Treatment |  |  |  |  |
| No | 162 (90.5%) | 53 (32.7%) | 109 (67.3%) | Referent |
| Yes | 17 (9.5%) | 7 (41.2%) | 10 (58.8%) | -8.5% (-32.9 to 16.0) |
| Cardiovascular Disease |  |  |  |  |
| No | 132 (73.7%) | 46 (34.8%) | 86 (65.2%) | Referent |
| Yes | 47 (26.3%) | 14 (29.8%) | 33 (70.2%) | 5.1% (-10.3 to 20.5) |
| Hypertension |  |  |  |  |
| No | 65 (36.3%) | 17 (26.2%) | 48 (73.8%) | Referent |
| Yes | 114 (63.7%) | 43 (37.7%) | 71 (62.3%) | -11.6% (-25.5 to 2.3) |
| COPD |  |  |  |  |
| No | 168 (93.9%) | 57 (33.9%) | 111 (66.1%) | Referent |
| Yes | 11 (6.1%) | 3 (27.3%) | 8 (72.7%) | 6.7% (-20.6 to 33.9) |
| Chronic Respiratory Disease |  |  |  |  |
| No | 92 (51.4%) | 38 (41.3%) | 54 (58.7%) | Referent |
| Yes | 87 (48.6%) | 22 (25.3%) | 65 (74.7%) | 16.0% (2.4 to 29.6) |
|  |  |  |  |  |

**Supplement 5. Mean days to infusion by month**
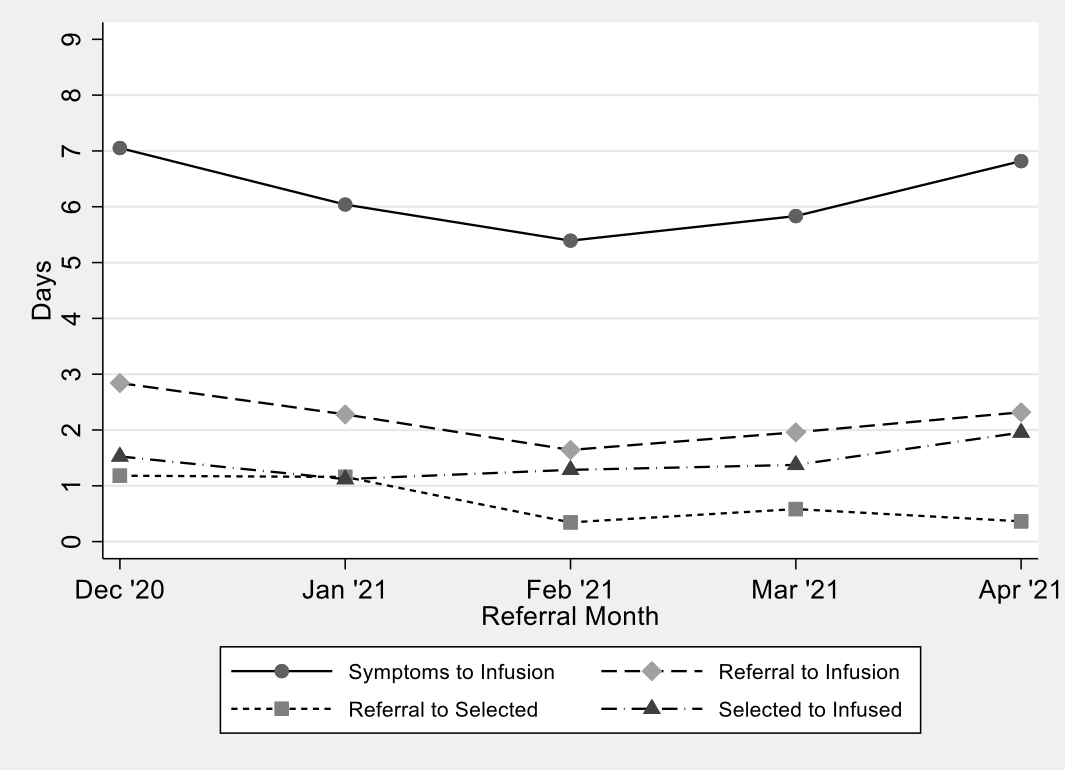

Supplement: ofad665_Supplementary_Data [file ofad665_supplementary_data.docx]
